## [Decision Letter · Decision Letter 0]

21 Feb 2025

Dear Dr. Li,

Thank you for submitting your manuscript to PLOS ONE. After careful consideration, we feel that it has merit but does not fully meet PLOS ONE’s publication criteria as it currently stands. Therefore, we invite you to submit a revised version of the manuscript that addresses the points raised during the review process.

We look forward to receiving your revised manuscript.

Kind regards,

Godwin Banafo Akrong, Ph.D.

Academic Editor

PLOS ONE

Journal Requirements:

https://journals.plos.org/plosone/s/file?id=ba62/PLOSOne_formatting_sample_title_authors_affiliations.pdf ..

4. In the online submission form you indicate that your data is not available for proprietary reasons and have provided a contact point for accessing this data. Please note that your current contact point is a co-author on this manuscript. According to our Data Policy, the contact point must not be an author on the manuscript and must be an institutional contact, ideally not an individual. Please revise your data statement to a non-author institutional point of contact, such as a data access or ethics committee, and send this to us via return email. Please also include contact information for the third party organization, and please include the full citation of where the data can be found.

Reviewers' comments:

Reviewer's Responses to Questions

**Comments to the Author**

1. Is the manuscript technically sound, and do the data support the conclusions?

Reviewer #1: Partly

Reviewer #2: Partly

2. Has the statistical analysis been performed appropriately and rigorously?

Reviewer #1: N/A

Reviewer #2: N/A

3. Have the authors made all data underlying the findings in their manuscript fully available?

Reviewer #1: Yes

Reviewer #2: No

4. Is the manuscript presented in an intelligible fashion and written in standard English?

Reviewer #1: No

Reviewer #2: Yes

Reviewer #1: 1.In the introduction section, please provide a reference for the sentence: “In China, due to the long-term influence of Confucian culture that prioritizes family, parents attach great importance to family ties and kinship and have a stronger sense of responsibility in raising children”.

2.In the inclusion criteria, “parenting a child aged ＜18 years” should be changed to “parenting at least a child aged ＜18 years”. The expression of “at a high risk of attrition” is not clear. Please clarify.

3.The participants were recruited using purposive sampling with maximum variation to ensure sample diversity in terms of education level and stage of cancer from 09/05/2024 to 30/06/2024. Yet you didn’t provide information about stage of cancer about your participants in your manuscript.

4.The sample size was determined by data saturation when no new theme emerged from the interviews12. After interviewing 13 participants, considerable data were repeated, which meant that data saturation had been reached. thus, data collection was halted. In this section, you mentioned data saturation, yet the criteria of judging that is repetition of themes and subthemes instead of data. Please clarify. Also, please pay attention to the format of writing as “thus” here should be “Thus”.

5.Researcher is considered as tool in qualitative study. In the data collection section, please provide more information about the researcher who conduct the interview, for example age, gender, occupation, etc. Also please provide the abbreviated name of the researcher who conduct the interview. It is also not clear to me whether you interview the couple one by one separately or together which is important to the study.

6.“When the interviewee's data repeatedly appears and there are no new themes in the data analysis, it is considered that the data is saturated, and the interview is stopped 14.” This sentence is a bit repetition of the previous sentence on saturation.

7.About the pre interview, it is better to mention what modifications were made afterwards.

8.One of the interview question is “What was your spouse's response after you told her/him about your concerns about the child?” Do you have male cancer patient in your study?

9.In the data analysis section, please be clear who did which part. The expression of one researcher or two researchers or third researcher etc. is not clear to me. I am not sure whether you need selective coding in a thematic analysis. Also, did you analyze the couple’s data together or how did you deal with it?

10.Please give the name of the guideline you adhere to when reporting the study and also check whether it is adhered to in the manuscript.

11.Name of table one should appear above the table instead of below the table.

12.Regarding your themes and subthemes, is it possible to provide more in depth analysis for some subthemes? I see many quotes put together and I think it is better to sort it out into different perspective if you consider all of them are different and necessary.

13.In the section of “Subtheme 2: proactive guidance of negative emotions”, some of the quotes are not quite supportive of the subthemes, please check again. For example:

After I fell ill, my husband was under a lot of pressure. Sometimes when my

younger soncried, my husband would feel irritableandhewould scold thechild.Idon't

blame him, I understand him. (P3)

I am concerned about the financial issues of my family. My husband and I will

discuss it, and he always come up with solutions to help me not worry. After discussing with my husband, I no longer worry as much as before. (P12)

14. The Subtheme communication concealment to avoid increasing parenting pressure looks a bit similar to the Subthem avoidance of communication to protect parenting emotions. Please reconsider and clarify.

15.In the discussion section, more explanation of why the findings are like this is needed . Also, please provide some cultural explanation of your findings because you mentioned about unique cultural background in China in the introduction section. Also how the finding is meaningful internationally?

16. There are some language issues such as “However, I don't have much energy to guide her (sigh) for my illness!” or “The intent is to avoid increasing the partner's psychological pressure, leading to the individual enduring their discomfort alone.”etc. Please read the manuscript carefully and revise accordingly.

Reviewer #2: Thank you for inviting me to review this manuscript. We believe this is a highly significant topic, focusing on communication about parenting among young breast cancer couples.

Throughout the paper, the terminology used to describe the research subjects varies, including terms such as patients, young breast cancer couples, and parents. This inconsistency may cause confusion for the reader and make it more difficult to fully grasp the research content. Additionally, some sections would benefit from further clarification, as certain parts are challenging to follow in terms of logical flow. A thorough review of the manuscript is encouraged to enhance consistency and improve readability.

Introduction

The introductory section references relevant studies; however, the discussion on prior research concerning communication between cancer patients and their spouses is somewhat brief. Expanding on the research findings and identifying gaps in previous studies is recommended, as this will also help clarify the research objectives.

The epidemiological description of breast cancer at the beginning of the Introduction is too much information, so I suggest you focus on what you want to assert.

It is difficult to understand the link between lower breast cancer incidence rates, delayed childbearing age, two or more children policy, and the upbringing of minors with breast cancer. We recommend that you cite the supporting papers and explain them clearly.

The section describing Chinese culture could be made more accessible to readers by including a comparison with other regions or countries and supporting the discussion with relevant citations or other evidence.

It may be a leap of logic to conclude that the negative effects of depression hinder rehabilitation and quality of life. Based on the aforementioned statements, it is unlikely that it is an impediment to rehabilitation. In addition, please present any articles that support this finding or consider removing the sentence “It can be~”.

Methods.

Providing a detailed description of the research methods enhances the scientific rigor of qualitative research. It is recommended to elaborate on the methods, as the current simplified description raises questions regarding its clarity and comprehensiveness.

Please explain the rationale for setting the participant's age as a criterion for inclusion in the study.

Please provide a detailed description of the random sampling procedure.

It should be clearly stated that both the participants and their spouses are included in the study. Since the study participants are patients and their spouses, the total number of participants should also be adjusted accordingly.

The relationship between the interviewer and the participant should be described. In qualitative research, reflexivity should be considered, as the researcher's background and perspective are integral to the research process, with the researcher themselves being considered an instrument of the study.

The description of when, where, and how the interviews were conducted is currently brief. Providing more detailed information will enhance the credibility of the paper.

Two terms are used: "interview outline" and "interview guide." I recommend unifying these terms. Alternatively, if they are intended to be used differently, please clarify the distinction between them.

Please provide a detailed description of the procedure for random sampling.

It should be explicitly stated that both the participants and their spouses are included in the study.

Since the study involves patients and their spouses, the total number of participants should also be revised accordingly.

The relationship between the interviewer and the participant should be clarified. In qualitative research, reflexivity is important, as the researcher's background and perspective are considered integral to the research process, with the researcher themselves being viewed as an instrument of the study.

The description of when, where, and how the interviews were conducted is currently brief. Providing more detailed information will enhance the credibility of the paper.

Two terms are used: "interview outline" and "interview guide." I recommend standardizing these terms, unless there is an intentional distinction between them that should be explained.

Please provide the name of the reporting guideline for the paper and add supporting references.

Results

Although the discussion states that the study is a dyadic exploration of the communication experiences of young breast cancer couples raising their minor children, the results do not include a dyadic analysis. In order to use the term "dyadic," the results should reflect the analysis of the couple pairs.

Some terms are defined within the results (e.g., negative communication, avoidance of communication, etc.). It is recommended to provide the relevant literature that defines these terms.

The results not only describe communication experiences but also indicate future communication intentions. Since "I am going to say it" refers to a future intention, it is unclear whether the participant actually expressed this intention. This distinction should be addressed in the discussion.

The distinction between subthemes 1 and 2 of Theme 2 is unclear. Please reconsider whether it is necessary to separate these two subthemes.

Discussion

The discussion should interpret the results of this study in relation to prior studies. This paper presents minimal engagement with existing literature, making it difficult to establish a clear logical and scientific foundation. Numerous studies have examined communication among couples facing cancer. Incorporating the findings of these studies could provide a deeper understanding by examining the relationships and differences between communication about parenting and communication about cancer. A thorough review of the entire document is recommended to ensure textual consistency, as some explanations are insufficient or lack logical clarity.

**Do you want your identity to be public for this peer review?** For information about this choice, including consent withdrawal, please see our For information about this choice, including consent withdrawal, please see our Privacy Policy .

Reviewer #1: No

Reviewer #2: No

While revising your submission, please upload your figure files to the Preflight Analysis and Conversion Engine (PACE) digital diagnostic tool, https://pacev2.apexcovantage.com/ . PACE helps ensure that figures meet PLOS requirements. To use PACE, you must first register as a user. Registration is free. Then, login and navigate to the UPLOAD tab, where you will find detailed instructions on how to use the tool. If you encounter any issues or have any questions when using PACE, please email PLOS at . PACE helps ensure that figures meet PLOS requirements. To use PACE, you must first register as a user. Registration is free. Then, login and navigate to the UPLOAD tab, where you will find detailed instructions on how to use the tool. If you encounter any issues or have any questions when using PACE, please email PLOS at figures@plos.org . Please note that Supporting Information files do not need this step.. Please note that Supporting Information files do not need this step.

---

## [Author Response · Author response to Decision Letter 1]

11 Apr 2025

Dear Professors,

Thank you for your letter and the opportunity to revise our paper on " Navigating parenting challenges: a qualitative study of communication experiences among young Chinese couples facing breast cancer while raising underage children”. The suggestions offered by the reviewers have been immensely helpful, and we also appreciate the insightful comments on revising the manuscript.

We have tabulated the reviewer’s comments immediately after this letter and responded to them individually, indicating exactly how we addressed each concern or problem and describing the changes we have made. The revisions have been approved by all authors. The changes are marked in red in the paper as you requested, and the revised manuscript was uploaded and submitted to the author centre.

We hope the revised manuscript is deemed suitable for the Plos One, and we are happy to consider further revisions if necessary. We look forward to a favourable reply, and we thank you for your continued interest in our research. If we do not meet the modification criteria, please give us another opportunity to communicate and make modifications.

---

## [Decision Letter · Decision Letter 1]

12 May 2025

Dear Dr. Li,

Thank you for submitting your manuscript to PLOS ONE. After careful consideration, we feel that it has merit but does not fully meet PLOS ONE’s publication criteria as it currently stands. Therefore, we invite you to submit a revised version of the manuscript that addresses the points raised during the review process.

We look forward to receiving your revised manuscript.

Kind regards,

Godwin Banafo Akrong, Ph.D.

Academic Editor

PLOS ONE

Reviewers' comments:

Reviewer's Responses to Questions

**Comments to the Author**

Reviewer #1: All comments have been addressed

Reviewer #2: (No Response)

2. Is the manuscript technically sound, and do the data support the conclusions?

Reviewer #1: Yes

Reviewer #2: Partly

3. Has the statistical analysis been performed appropriately and rigorously?

Reviewer #1: N/A

Reviewer #2: N/A

4. Have the authors made all data underlying the findings in their manuscript fully available?

Reviewer #1: Yes

Reviewer #2: No

5. Is the manuscript presented in an intelligible fashion and written in standard English?

Reviewer #1: Yes

Reviewer #2: No

Reviewer #1: The revision is good and it address all of my issues. I recommend the authors to pay attention to some of the language issues as well in the manuscript.

Reviewer #2: １．Introduction

Q1.　In the Introduction, previous studies on communication between cancer patients and their spouses are reviewed, and the research questions are presented. However, the purpose of the study is not clearly stated. Please clearly articulate the purpose of the study in the main text.

Additionally, what distinguishes the general parenting concerns of cancer patients from those specific to breast cancer patients? The rationale for focusing specifically on breast cancer patients in this study should be clearly explained.

Q2.　“Compared to the　emphasis on cultivating children's independence in other culture[7], “in China, due to the long-term influence of Confucian culture that prioritizes family, parents attach great importance to family ties and kinship and have a stronger sense of responsibility in raising children.

This seems to involve a logical leap. While I understand that Chinese families place great importance on child-rearing, other cultures also highly value family and children. In general, when mothers are unable to care for their children due to cancer, it is reasonable to expect that they would experience significant concern and anxiety. A more nuanced and culturally sensitive explanation would strengthen this argument.

２．Method

Q3. (3) parenting at least a child aged ＜ 18 years

The current phrasing is unclear and may be difficult for readers to interpret. If the intended meaning is that the individual is parenting at least one child under 18 years of age, a clearer and more natural expression should be used.

Q4. (1) those who are aware of their spouse's condition;

It is unclear who the term “spouse” refers to in this context. If it refers to the breast cancer patient, the wording should be revised to avoid confusion.

３．Discussion

Q5.　In this study, we have interviewed 13 patients.

You are conducting interviews with 13 couples who are breast cancer patients, right? Please revise the text.

Q6. The influence of Chinese culture might be somewhat overstated in this analysis. Many of the findings in this study appear to reflect experiences and emotional responses that are common across cultures. In particular, patients’ and their spouses’ perceptions and emotional reactions to cancer, as well as their love and concern for their children, are likely to share significant commonalities regardless of cultural background.

Moreover, previous studies have shown that some cancer patients often suppress their own emotions and avoid sharing them with family members in an effort to protect their loved ones. It is recommended that the discussion engage more thoroughly with the existing literature on communication among cancer patients, their spouses, and families. In light of these considerations, it would be appropriate to revise the discussion section.

Additionally, for patients and spouses who are coping with economic insecurity, caregiving responsibilities, childcare, and household burdens, it is worth questioning whether communication support alone is sufficient to address these challenges. It may be worth considering whether adequate formal and informal support systems—both public and private—exist to assist families facing such difficulties. It may be helpful to note the need to consider broader structural and social support issues.

**Do you want your identity to be public for this peer review?** For information about this choice, including consent withdrawal, please see our For information about this choice, including consent withdrawal, please see our Privacy Policy .

Reviewer #1: No

Reviewer #2: No

While revising your submission, please upload your figure files to the Preflight Analysis and Conversion Engine (PACE) digital diagnostic tool, https://pacev2.apexcovantage.com/ . PACE helps ensure that figures meet PLOS requirements. To use PACE, you must first register as a user. Registration is free. Then, login and navigate to the UPLOAD tab, where you will find detailed instructions on how to use the tool. If you encounter any issues or have any questions when using PACE, please email PLOS at . PACE helps ensure that figures meet PLOS requirements. To use PACE, you must first register as a user. Registration is free. Then, login and navigate to the UPLOAD tab, where you will find detailed instructions on how to use the tool. If you encounter any issues or have any questions when using PACE, please email PLOS at figures@plos.org . Please note that Supporting Information files do not need this step.. Please note that Supporting Information files do not need this step.

---

## [Author Response · Author response to Decision Letter 2]

4 Sep 2025

Dear Professors,

Thank you for your letter and the opportunity to revise our paper again. The suggestions offered by the reviewers have been immensely helpful, and we also appreciate the insightful comments on revising the manuscript.

We have tabulated the reviewer’s comments immediately after this letter and responded to them individually, indicating exactly how we addressed each concern or problem and describing the changes we have made. The revisions have been approved by all authors. The changes are marked in red in the paper as you requested, and the revised manuscript was uploaded and submitted to the author centre.

We hope the revised manuscript is deemed suitable for the Plos One, and we are happy to consider further revisions if necessary. We look forward to a favourable reply, and we thank you for your continued interest in our research. If we do not meet the modification criteria, please give us another opportunity to communicate and make modifications.

---

## [Decision Letter · Decision Letter 2]

9 Dec 2025

Dear Dr. Li,

We look forward to receiving your revised manuscript.

Kind regards,

Godwin Banafo Akrong, Ph.D.

Academic Editor

PLOS ONE

Journal Requirements:

Reviewers' comments:

Reviewer's Responses to Questions

**Comments to the Author**

Reviewer #1: All comments have been addressed

Reviewer #3: All comments have been addressed

Reviewer #4: All comments have been addressed

2. Is the manuscript technically sound, and do the data support the conclusions?

Reviewer #1: Yes

Reviewer #3: Yes

Reviewer #4: Yes

3. Has the statistical analysis been performed appropriately and rigorously?

Reviewer #1: N/A

Reviewer #3: Yes

Reviewer #4: N/A

4. Have the authors made all data underlying the findings in their manuscript fully available?

Reviewer #1: Yes

Reviewer #3: Yes

Reviewer #4: Yes

5. Is the manuscript presented in an intelligible fashion and written in standard English?

Reviewer #1: Yes

Reviewer #3: Yes

Reviewer #4: Yes

Reviewer #1: I am satisfied with the revision and I have no further comments. I think with some proofreading, the manuscript is ready for publication.

Reviewer #3: This manuscript presents a longitudinal qualitative study exploring the expectations of Ghanaian women with metastatic breast cancer (MBC) regarding systemic treatment outcomes. It is an important and timely topic, particularly in the context of limited palliative oncology infrastructure in low- and middle-income countries (LMICs). The authors have demonstrated commendable effort in data collection and interpretation.

The study offers valuable insights into the mismatch between patients’ expectations (often curative) and the clinical reality of MBC as an incurable condition. The findings have clear implications for communication, counseling, and policy in oncology care in Ghana and similar contexts.

Reviewer #4: While the manuscript is strong, the following points could enhance its impact, clarity, and scholarly contribution

1. Speaking about Chinese culture in the discussion could you strengthen the discussion with deeper culture nuanced, this would bring a nuanced analysis from being culturally specific to being a culturally informed contribution to the global literature on cancer couple communication

2. Result section: Suggestion for Sub-theme 2.1 ("instrumental support" and emotional avoidance). This is a powerful finding, is it possible to get some quotes hat shows the husband's practical action and the wife's perception of emotional distance in the same interaction.

**Do you want your identity to be public for this peer review?** For information about this choice, including consent withdrawal, please see our For information about this choice, including consent withdrawal, please see our Privacy Policy .

Reviewer #1: No

Reviewer #3: **Yes:** Kofi Boamah MensahKofi Boamah Mensah

Reviewer #4: No

---

## [Author Response · Author response to Decision Letter 3]

22 Dec 2025

To reviewer #4:

Dear professor, thank you very much for your valuable guidance. The suggestions offered by you have been immensely helpful, and we also appreciate the insightful comments on revising the manuscript.

We have add information at Page 17-18 to bring a nuanced analysis from being culturally specific to being a culturally informed contribution to the global literature on cancer couple communication. We have added more quotes at Page 13 to show the husband's practical action and the wife's perception of emotional distance in the same interaction. Thanks again.

---

## [Decision Letter · Decision Letter 3]

12 Jan 2026

Dear Dr. Li,

Thank you for submitting your manuscript to PLOS ONE. After careful consideration, we feel that it has merit but does not fully meet PLOS ONE’s publication criteria as it currently stands. Therefore, we invite you to submit a revised version of the manuscript that addresses the points raised during the review process.

I encourage you to address the comments raised by Reviewer #4 and make the necessary revisions. I look forward to reviewing your revised manuscript.

We look forward to receiving your revised manuscript.

Kind regards,

Godwin Banafo Akrong, Ph.D.

Academic Editor

PLOS One

Journal Requirements:

Reviewers' comments:

Reviewer's Responses to Questions

**Comments to the Author**

Reviewer #1: All comments have been addressed

Reviewer #4: All comments have been addressed

2. Is the manuscript technically sound, and do the data support the conclusions?

Reviewer #1: Yes

Reviewer #4: Yes

3. Has the statistical analysis been performed appropriately and rigorously?

Reviewer #1: N/A

Reviewer #4: Yes

4. Have the authors made all data underlying the findings in their manuscript fully available?

Reviewer #1: Yes

Reviewer #4: Yes

5. Is the manuscript presented in an intelligible fashion and written in standard English?

Reviewer #1: Yes

Reviewer #4: Yes

Reviewer #1: I am satisfied with the revision. There is no more suggestions for revision. I think this manuscript is now ready for publication.

Reviewer #4: Thank you for addressing my initial comments : However, I have further concerns:

1. Can the authors clarify why they chose to conduct the study from the Yibin Second People’s Hospital?

2. The authors provided enough demographic information on the participants, however, in reporting the quotes made by the participants the authors did not support them with these demographics to help readers appreciate the findings. The authors can report it as follows (S11, 40 years, staff) OR (P11, 35 years, 2 children, Period III)

3. The implications of the study should clearly be presented by the authors.

4. Although the authors mention purposive sampling with maximum variation, they did not provide detailed information on how diversity in education level, cancer stage, and other demographic factors were ensured during participant recruitment.

5. The authors state that data saturation was achieved after interviewing 13 couples, however, can the authors provide a clear explanation of how saturation was determined. Including specific criteria.

6. Since the authors mention that the interviewer played dual roles as an “insider” and “outsider,” can the authors elaborate on how potential bias from the interviewer’s prior relationship with patients was mitigated?

7. Can the authors provide more information on how codes were developed and refined, as well as the process of resolving discrepancies between researchers. This is to help address the issues of transparency and replicability.

**Do you want your identity to be public for this peer review?** For information about this choice, including consent withdrawal, please see our For information about this choice, including consent withdrawal, please see our Privacy Policy .

Reviewer #1: No

Reviewer #4: No

---

## [Author Response · Author response to Decision Letter 4]

13 Feb 2026

Dear Professor, Godwin Banafo Akrong,

Thank you for your letter and the opportunity to revise our paper again. The suggestions offered by Reviewer #4 have been immensely helpful, and we also appreciate the insightful comments on revising the manuscript.

We have tabulated the reviewer’s comments immediately after this letter and responded to them individually, indicating exactly how we addressed each concern or problem and describing the changes we have made. The revisions have been approved by all authors. The changes are marked in red in the paper, and the revised manuscript was uploaded and submitted to the author centre.

We hope the revised manuscript is deemed suitable for the Plos One, and we are happy to consider further revisions if necessary. We look forward to a favourable reply, and we thank you for your continued interest in our research. If we do not meet the modification criteria, please give us another opportunity to communicate and make modifications.

Sincerely,

LiYingchun

---

## [Decision Letter · Decision Letter 4]

9 Mar 2026

Navigating parenting challenges: a qualitative study of communication experiences among young Chinese couples facing breast cancer while raising underage children

PONE-D-24-43554R4

Dear Dr. Li,

We’re pleased to inform you that your manuscript has been judged scientifically suitable for publication and will be formally accepted for publication once it meets all outstanding technical requirements.

Kind regards,

Godwin Banafo Akrong, Ph.D.

Academic Editor

PLOS One

Additional Editor Comments (optional):

Reviewers' comments:

Reviewer's Responses to Questions

**Comments to the Author**

Reviewer #1: All comments have been addressed

Reviewer #4: All comments have been addressed

2. Is the manuscript technically sound, and do the data support the conclusions?

Reviewer #1: Yes

Reviewer #4: Yes

3. Has the statistical analysis been performed appropriately and rigorously?

Reviewer #1: N/A

Reviewer #4: N/A

4. Have the authors made all data underlying the findings in their manuscript fully available?

Reviewer #1: Yes

Reviewer #4: Yes

5. Is the manuscript presented in an intelligible fashion and written in standard English?

Reviewer #1: Yes

Reviewer #4: Yes

Reviewer #1: 1.Please be clear in table 1 about whose age and occupation.

2.Please mention about recruitment together not seperate in difference location of the paragraph.

3. There are some minor grammar issues that such "The sample size was determined by data saturation, which was achieved when no new themes emerged from the interviews and the assess method from Guest

[25]."

Reviewer #4: I don't have further comments. All required questions have been answered and that all responses meet formatting specifications.

**Do you want your identity to be public for this peer review?** For information about this choice, including consent withdrawal, please see our For information about this choice, including consent withdrawal, please see our Privacy Policy .

Reviewer #1: No

Reviewer #4: No

---

## [Editor Report · Acceptance letter]

PONE-D-24-43554R4

PLOS One

Dear Dr. Li,

I'm pleased to inform you that your manuscript has been deemed suitable for publication in PLOS One. Congratulations! Your manuscript is now being handed over to our production team.

Kind regards,

on behalf of

Dr. Godwin Banafo Akrong

Academic Editor

PLOS One