# Supplementary material for: Navigating parenting challenges: A qualitative study of communication experiences among young Chinese couples facing breast cancer while raising underage children
Source: PLoS One. 2026 Apr 2;21(4):e0345543. doi: 10.1371/journal.pone.0345543 (PMC13046115; doi:10.1371/journal.pone.0345543)
Supplement: S1 Text — (DOCX) [file pone.0345543.s002.docx]

**S1 Text. Interview Transcript Text**

**Participant Code Key:** P = Patient; S = Spouse. The number represents the specific couple dyad (e.g., P01 and S01 are a couple).

**Couple 01**

Researcher (to P01): Since falling ill, have you been concerned about your child? What are you concerned about?
P01: Very much. I'm most worried that my emotions will affect her. I'm sometimes too tired from treatment to be patient with her, and I feel terribly guilty afterwards. I also worry that seeing me sick will scare her and leave psychological scars and my illness would affect my child's learning.

Researcher (to P01): Would you tell your husband about these concerns regarding your child? Why?
P01: Yes, I would. Because he is my husband and the child's father. I feel we have a responsibility to face these problems together; I can't carry them alone. Telling him makes me feel better, like I'm not fighting this battle by myself.

Researcher (to P01): What was his response after you told him? Did he communicate with you?
P01: He usually tells me not to blame myself so much. Then he'll say, "Let's think together about what to do." For example, we might plan to take our daughter to the park on the weekend to spend special time with her, or he'll proactively take over reading bedtime stories so I can rest. This kind of communication makes me feel like we're finding solutions together.

Researcher (to P01): Did his responses affect you? How did that make you feel?
P01: It affected me greatly. I feel a sense of relief, like the pressure is being shared. His understanding and actions make me feel warm and supported, and it gives me more confidence in my role as a mother.

Researcher (to S01): Since your wife fell ill, have you been concerned about your child? What are you concerned about?
S01: Yes, I'm concerned. I worry about our child's schoolwork because my wife used to manage it more, and now she needs rest—I'm afraid their grades might slip. I'm even more concerned about our child's emotions, whether they can understand Mom's illness and if they are feeling sad or scared inside.

Researcher (to S01): Would you tell your wife about these concerns regarding your child? Why?
S01: I am selective about what I share. I'll immediately share good, positive news to make her happy. I will also share smaller worries. I don't want to hide things from her; she is the mother and has a right to know. But I have to be careful about how I do it.

Researcher (to S01): After you told her, what was her response? Did she communicate with you?

S01: She sometimes gets anxious, but more often, she discusses it with me. I think she was worrying too much.

Researcher (to S01): Did her responses affect you? How did that make you feel?
S01: It feels very good. It makes me feel like we are still a complete team. Even during this difficult time, we can still be responsible for our child together. Her suggestions often give me insight and make me feel less anxious.

**Couple 02**

Researcher (to P02): Are you concerned about your child since falling ill?

P02: When I think of my two children at night, I can't help but shed tears. I worry that I can't take care of them like I used to, worry about their future without a mother. (Crying)

P02: I am worried that my child will be mocked by his classmate for my illness. But I don't tell my husband and my child (with a bitter smile). I'm almost depressed now and I feel like I can't get out!

Researcher (to P02): Would you tell your spouse about your concerns about the child?

P02: Yes, I told my husband. I hope he can support me.

Researcher (to P02): What was your spouse's response after you told her/him about your concerns about the child? Did your spouse communicate with you?

P02: My husband comforted me and advised me not to overthink. He said that my illness will get better and my child will grow up.

S02: Since she was diagnosed, she has been worried about various things and she asked me if she's gone, will I do my best to raise the child. I told her that the medical technology is already mature now, and there are also many people suffering from this disease, which is just a common illness. I tried to alleviate her concerns.

Researcher (to S02): Are you concerned about your child since falling ill?

S02: I'm mainly worried my son is becoming withdrawn. He's been talking less lately.

Researcher (to S02): Would you tell your wife?

S03: I don't really dare to. She tends to overthink things. I can't put too much pressure on her anymore. I just need to endure the pressure alone. I don't tell her (my pressure), usually only say good things. I'm afraid if she knows, she'll dwell on it, blame herself, think it's because of her illness, and that will affect her mood and recovery. So I usually just try to spend more time playing with my child myself, trying to cheer him up.

**Couple 03**

S3: I have two children, the oldest is 7 years old and the youngest is 2 years old. (I work out of town) So my wife takes care of the children alone. It's not possible now. My wife and I have discussed that my parents in law will take care of the children.

P3: We discussed seeking help from my parents to take care of the child.

**Couple 04**

P04: I would tell him. But sometimes his reaction makes me feel worse.
Researcher: Can you give an example?

P04: I told him I was afraid I couldn't tutor the kids anymore. He just said, "It's fine, I'll tutor them from now on, you don't need to worry about it." The intention was right, but it sounded cold, like he was pushing me out of my role as a mother. I just wanted a little comfort, not just a direct "solution." He only does things silently, but never talks to me about my anxiety about children.

S04: I don't know how to comfort people. It's my responsibility to make money and take care of children. Leaving the crying to her.

P04: I will not tell him my concerns, I don't want to (cry), we have different opinions, and we argue as soon as we talk. Whenever we argue, it affects my emotions, and I don't want to suffer from emotional pain. I told him about my concerns about the child, he only looked at his phone and ignored me.

S04: I don’t want to talk about the topic with her.

**Couple 05**

P5: My youngest son is only 2 years old, and he also has a serious illness (crying). Now that I am suffering from this illness again, I cannot take good care of my son and cannot bear the burden for him (crying). I suffer from insomnia all night long, and sometimes I can't bear it anymore. I tell my husband to spend more time with my youngest son, and he tries his best to take care of him. Whenever he has free time, he plays with him.

S05: I would tell her. But I noticed that every time we talk about the child's problems, her emotions fluctuate wildly—she either cries or becomes very anxious. Later, I became somewhat afraid and simply stopped bringing things up. I try to report only good news, not bad, and handle everything myself before mentioning it.

**Couple 06**

P06: My husband has been accompanying me for treatment since I fell ill. He is now facing the cost of raising children and my treatment expenses, and he is under a lot of psychological pressure. My sister helped us to take care of my two daughters. I have a lot of concerns about my daughters. I'm worried that her classmates will laugh at her and worry about her studies when they know my illness at school (sighing). But I don't want to share my concerns with my husband. I'm worried about adding to his burden as he's already under a lot of pressure. When I feel uncomfortable, I just endure it myself.

S6: My wife is sick and I can't put too much pressure on her anymore. Everything is in my heart, I know how to do it, I won't tell her (my pressure), usually only say good things.

**Couple 07**

P7: My eldest daughter has dropped out of school, and I am worried that she will endure hardship. However, I don’t have much energy to guide her because of my illness!" (sigh). My son can't do this anymore. My husband and I have discussed we will arrange for the child a primary school in a county-level school. After the chemotherapy is over, I will accompany him to study.

S7: We have decided to arrange our son to a county-level school.

**Couple 08**

P08: I have been unable to take care of them well, and I cannot help but worry that others may not be able to take care of them. I will tell my husband that I want to do my best to be a good mother, but I can't do it! I feel helpless and painful! (Crying)

P08: I told him I was worried our child would be teased at school for having a bald mom. The next day, he went to pick up our child and specifically chatted with a few friends and parents, casually mentioning that "Mom's treatment is giving her hair a temporary rest, it will grow back even prettier." That action made me feel more supported than any words could have. It made communicating about solutions much smoother afterwards.

S08: I love my wife. I do everything to alleviate her anxiety. I won't tell her my concerns because I don't want to add to her worries and psychological pressure. This illness requires a good mood.

**Couple 09**

S09: Her reaction directly affects me. If I share a problem and she becomes very distraught, I feel instant regret, like I've added to her burden, and I become hesitant to share next time. If she can discuss it calmly with me, even if the problem isn't solved, I feel like the weight on my shoulders is halved because someone is sharing the load.

P9: I am sick now, and all kinds of expenses at home are weighing on my husband. I didn't tell him my concerns, I afraid of adding psychological burden to him.

**Couple 10**

P10: I don't share everything. For small things, like our child scraping their knee, I handle it myself. I only choose to share things I think he needs to know about or that we need to solve together. I don't want him to think I'm a huge burden.

S10: My wife is always worried that without her to take the children to school, their emotions will be affected. I arrange transportation for my child to school every day, I think her concerns are unnecessary.

**Couple 11**

S11: I haven't been with my child for a long time, I'm not very familiar with his habits. I have to ask my wife about many things, and I will exchange experiences and ideas with her. The only purpose is to reduce the impact on my son.

P11: He's not quite sure about the child's habits, I told him.

P11: I always worry about what will happen to the children when I'm gone. I tell him, but if I say it too many times, he will ignore me. He is very indifferent to me and never cares about my feelings.

S11: She told me countless times what to do with the children when she's gone, and I'm getting tired of hearing it. There's nothing to say. I feel powerless.

**Couple 12**

P12: Once, I cried and told him I dreamed our child couldn't find me. He didn't laugh at me or say "dreams aren't real." He just held me tight and said, "I know that dream scared you. I promise you I will do everything I can to make sure you're there to watch them grow up" In that moment, I didn't need logic; I needed that emotional connection and support.

S12: Just support her. I usually chat with her about children's affairs.

**Couple 13**

Researcher (to S13): Would you tell your spouse about your concerns about the child?

P13: My daughter is 3 years old. After I fell ill, my husband took the initiative to take care of my daughter. We discussed our experiences in taking care of my daughter and exchanged experiences with each other.

Researcher (to S13): How did your wife's responses affect you?

S13: I discuss my daughter's matter with her. When she thanks me for everything I do for the child and the family, I feel all the hard work is worth it. When she can calmly share her worries with me, I feel an incredible closeness and sense of trust between us. This positive feedback is a huge motivation for me to keep going.
