# Supplementary material for: Navigating parenting challenges: A qualitative study of communication experiences among young Chinese couples facing breast cancer while raising underage children
Source: PLoS One. 2026 Apr 2;21(4):e0345543. doi: 10.1371/journal.pone.0345543 (PMC13046115; doi:10.1371/journal.pone.0345543)
Supplement: Attachment — Submitted filename: Response to Reviewers.docx [file pone.0345543.s004.docx]

Prof. Godwin Banafo Akrong

Editor,

PLOS ONE

April 6, 2025

Subject: Revision and resubmission of manuscript / PONE-D-24-43554

Dear Professor, Godwin Banafo Akrong,

Thank you for your letter and the opportunity to revise our paper on " Navigating parenting challenges: a qualitative study of communication experiences among young Chinese couples facing breast cancer while raising underage children”. The suggestions offered by the reviewers have been immensely helpful, and we also appreciate the insightful comments on revising the manuscript.

We have tabulated the reviewer’s comments immediately after this letter and responded to them individually, indicating exactly how we addressed each concern or problem and describing the changes we have made. The revisions have been approved by all authors. The changes are marked in **red** in the paper as you requested, and the revised manuscript was uploaded and submitted to the author centre.

We hope the revised manuscript is deemed suitable for the Plos One, and we are happy to consider further revisions if necessary. We look forward to a favourable reply, and we thank you for your continued interest in our research. If we do not meet the modification criteria, please give us another opportunity to communicate and make modifications.

Sincerely,

| **Academic Editor Comments** | **Authors Respond*** | **Section/Page (P) Number** |
| --- | --- | --- |
| 1. Please ensure that your manuscript meets PLOS ONE's style requirements, including those for file naming. | Thank you for your reminder. We have adjusted the format according to PLOS ONE's style requirements. |  |
| 2. Please include a complete copy of PLOS’ questionnaire on inclusivity in global research in your revised manuscript. Our policy for research in this area aims to improve transparency in the reporting of research performed outside of researchers’ own country or community. The policy applies to researchers who have travelled to a different country to conduct research, research with Indigenous populations or their lands, and research on cultural artefacts. The questionnaire can also be requested at the journal’s discretion for any other submissions, even if these conditions are not met. Please find more information on the policy and a link to download a blank copy of the questionnaire here: https://journals.plos.org/plosone/s/best-practices-in-research-reporting. Please upload a completed version of your questionnaire as Supporting Information when you resubmit your manuscript. | Thank you for your reminder. We have upload a completed version of the questionnaire. In addition, we have added “Inclusivity in global research ” in the Methods section of the revised manuscript. | P8 |
| 3. We note that you have indicated that there are restrictions to data sharing for this study. For studies involving human research participant data or other sensitive data, we encourage authors to share de-identified or anonymized data. However, when data cannot be publicly shared for ethical reasons, we allow authors to make their data sets available upon request. For information on unacceptable data access restrictions, please see http://journals.plos.org/plosone/s/data-availability#loc-unacceptable-data-access-restrictions. | Thank you for highlighting this issue.  We have revised the data statement:  Data cannot be shared publicly because of ethical restrictions imposed by the Yibin Second People’s Hospital Ethics Committee. Data are available from the Yibin Second People’s Hospital Institutional Data Access/Ethics Committee (contact via ybeyyllwyh@126.com, Zip Code: 644000, Tel: 0831-8257719, Fax: 0831-8257719) for researchers who meet the criteria for access to confidential data. | Page 19, see in Data availability section |
| 4. In the online submission form you indicate that your data is not available for proprietary reasons and have provided a contact point for accessing this data. Please note that your current contact point is a co-author on this manuscript. According to our Data Policy, the contact point must not be an author on the manuscript and must be an institutional contact, ideally not an individual. Please revise your data statement to a non-author institutional point of contact, such as a data access or ethics committee, and send this to us via return email. Please also include contact information for the third party organization, and please include the full citation of where the data can be found. | Thank you for highlighting this issue.  We have revised the data statement:  Data cannot be shared publicly because of ethical restrictions imposed by the Yibin Second People’s Hospital Ethics Committee. Data are available from the Yibin Second People’s Hospital Institutional Data Access/Ethics Committee (contact via ybeyyllwyh@126.com, Zip Code: 644000, Tel: 0831-8257719, Fax: 0831-8257719) for researchers who meet the criteria for access to confidential data. | Page 19, see in Data availability section |
| **Reviewer #1 Comments** | **Authors Respond*** | **Section/Page (P) Number** |
| 1. In the introduction section, please provide a reference for the sentence: “In China, due to the long-term influence of Confucian culture that prioritizes family, parents attach great importance to family ties and kinship and have a stronger sense of responsibility in raising children” | Thank you for your insightful feedback, which has been instrumental in enhancing the quality of this manuscript. We have thoroughly reviewed and incorporated your suggestions to optimize the content. Below is a detailed response addressing each of your comments for your review.  Thank you for your advice. We have checked the literature carefully and added relevant references into the Introduction part of the revised manuscript.  “in China, due to the long-term influence of Confucian culture that prioritizes family, parents attach great importance to family ties and kinship and have a stronger sense of responsibility in raising children^[8, 9]^.”  Reference:  8. Chao, R.K., Beyond parental control and authoritarian parenting style: understanding Chinese parenting through the cultural notion of training. Child Dev, 1994. **65**(4): p. 1111-9.  9. Yang, J. and Y. Zheng, Links Between Perceptions of Successes, Problems and Health Outcomes Among Adult Chinese Children: The Mediating Role of Perceptions of Parents' Feelings and Intergenerational Relationships. Front Psychol, 2019. **10**: p. 2551. | Page 2, see in “Introduction” section, paragraph 2 |
| 2. In the inclusion criteria, “parenting a child aged ＜18 years” should be changed to “parenting at least a child aged ＜18 years”. The expression of “at a high risk of attrition” is not clear. Please clarify. | Thank you very much for your guidance. We have revised accordingy:  The potential participants were patients who met the following inclusion criteria: (1) age 20-44 years old; (2) pathological diagnosis of breast cancer, and knowing their condition; (3) parenting at least a child aged ＜18 years; (4) physically able to participate; and (5) willing to provide informed consent to participate in this study. The inclusion criteria for spouses were: (1) those who are aware of their spouse's condition; (2) informed consent and voluntary participation in this study. Patients or their spouses with cognitive or mental disorders that inhibit cooperation, with serious comorbidities were excluded. | Page 5 |
| 3. The participants were recruited using purposive sampling with maximum variation to ensure sample diversity in terms of education level and stage of cancer from 09/05/2024 to 30/06/2024. Yet you didn’t provide information about stage of cancer about your participants in your manuscript. | We sincerely thank the reviewer for careful reading. We have added the information of participants’ stage of cancer. | Page 9, Table 1. |
| 4. The sample size was determined by data saturation when no new theme emerged from the interviews^12^. After interviewing 13 participants, considerable data were repeated, which meant that data saturation had been reached. thus, data collection was halted. In this section, you mentioned data saturation, yet the criteria of judging that is repetition of themes and subthemes instead of data. Please clarify. Also, please pay attention to the format of writing as “thus” here should be “Thus”. | We sincerely thank the reviewer for careful reading. As suggested by the reviewer, we have clarified the information:  The sample size was determined by data saturation when no new theme emerged from the interviews. | Page 5, paragraph 1 |
| 5. Researcher is considered as tool in qualitative study. In the data collection section, please provide more information about the researcher who conduct the interview, for example age, gender, occupation, etc. Also please provide the abbreviated name of the researcher who conduct the interview. It is also not clear to me whether you interview the couple one by one separately or together which is important to the study. | Thank you for your valuable feedback. We appreciate your feedback regarding the data collection section. We have added detailed description to enhance the reliability of the manuscript:  The interview was conducted in the conversation room of the breast department. Face-to-face interviews were individually conducted by a researcher (YYM) with extensive experience in qualitative interviewing. The researcher is the head nurse with master’s degree (46 years old, associate chief nurse, female), and had been working in breast department for over 10 years.  In this study, patients and their spouses were interviewed separately. | Page 5-6 |
| 6.“When the interviewee's data repeatedly appears and there are no new themes in the data analysis, it is considered that the data is saturated, and the interview is stopped 14.” This sentence is a bit repetition of the previous sentence on saturation. | Thank you for your guidance. In order to enhance the readability of the article, we have removed the sentence. |  |
| 7. About the pre interview, it is better to mention what modifications were made afterwards. | Thank you for your advice. we have added the modifications were made in the section of interview outline.  Several modifications on inquiry method were made according to the interviews to form the formal interview outline. For example, we modified patient’s interview outline“After falling ill, how does your spouse communicate with you about their thoughts on child parenting?” to “Would you tell your husband about your concerns about your child? Why?”. | Page 5, see in “Data collection” section. |
| 8. One of the interview question is “What was your spouse's response after you told her/him about your concerns about the child?” Do you have male cancer patient in your study? | We sincerely thank the reviewer for careful reading. Only female breast cancer patient. We have revised the interview outline. | Page 5-6, see in the section of “Data collection”. |
| 9. In the data analysis section, please be clear who did which part. The expression of one researcher or two researchers or third researcher etc. is not clear to me. I am not sure whether you need selective coding in a thematic analysis. Also, did you analyze the couple’s data together or how did you deal with it? | Thank you for your valuable guidance. We have added detailed information to describe the data analysis process.  We have re-analyzed the data from "dyadic" perspective, and refined the themes. | Page 7, see in Data analysis section. |
| 10. Please give the name of the guideline you adhere to when reporting the study and also check whether it is adhered to in the manuscript. | Thank you for your advice. We have added the reporting guideline for the paper and add supporting references.  Reporting was performed in accordance with the Consolidated Criteria for Reporting Qualitative Research (COREQ) reporting guidelines. | Page 4, see in “Design and setting” section. |
| 11. Name of table one should appear above the table instead of below the table. | We sincerely thank the reviewer for careful reading. As suggested by the reviewer, we have revised. | Page 9, Table 1. |
| 12. Regarding your themes and subthemes, is it possible to provide more in depth analysis for some subthemes? I see many quotes put together and I think it is better to sort it out into different perspective if you consider all of them are different and necessary. | Thank you for your advice. We have provided more in depth analysis to the themes. | Section of Results, Page 10-14 |
| 13. In the section of “Subtheme 2: proactive guidance of negative emotions”, some of the quotes are not quite supportive of the subthemes, please check again. For example: After I fell ill, my husband was under a lot of pressure. Sometimes when my younger soncried, my husband would feel irritable and he would scold the child.I don't blame him, I understand him. (P3) I am concerned about the financial issues of my family. My husband and I will discuss it, and he always come up with solutions to help me not worry. After discussing with my husband, I no longer worry as much as before. (P12) | Thank you for your guidance. We have checked and analyzied the data, and revised the quotes.  **We have removed the unsuitable quote** “After I fell ill, my husband was under a lot of pressure. Sometimes when my younger soncried, my husband would feel irritable and he would scold the child.I don't blame him, I understand him. (P3)”  **We have clarified the quote** “I am concerned about the financial issues of my family. My husband and I will discuss it, and he always come up with solutions to help me not worry. After discussing with my husband, I no longer worry as much as before. (P12)”. **The correct quote is** “I am concerned about the parenting ability of my husband, especially the financial issues of parenting child. My husband and I discussed it, and he always come up with solutions to help me not worry. After discussing with my husband, I no longer worry as much as before. (P12)” | Page 11-12 |
| 14. The Subtheme communication concealment to avoid increasing parenting pressure looks a bit similar to the Subthem avoidance of communication to protect parenting emotions. Please reconsider and clarify. | We sincerely appreciate the valuable comments. As suggested by the reviewer, we have re-analysis the data and have revised sub-theme 1 of Theme 2.  We have revised “Subtheme 1: avoidance of communication to protect parenting emotions” to “Subtheme 1: Avoid communication to prevent emotional fluctuations”. | Page 12 |
| 15. In the discussion section, more explanation of why the findings are like this is needed . Also, please provide some cultural explanation of your findings because you mentioned about unique cultural background in China in the introduction section. Also how the finding is meaningful internationally? | Thanks for your excellent suggestion. We have added more information regarding why the findings are like this and provided cultural explanation. | Page 14-18 |
| 16. There are some language issues such as “However, I don't have much energy to guide her (sigh) for my illness!” or “The intent is to avoid increasing the partner's psychological pressure, leading to the individual enduring their discomfort alone.”etc. Please read the manuscript carefully and revise accordingly. | Thank you for your careful checks. We sincerely appreciate the valuable comments. We have proofread the manuscript and have revised. | Page 11-12 |
| **Reviewer #2 Comments**  Thank you for inviting me to review this manuscript. We believe this is a highly significant topic, focusing on communication about parenting among young breast cancer couples.  Throughout the paper, the terminology used to describe the research subjects varies, including terms such as patients, young breast cancer couples, and parents. This inconsistency may cause confusion for the reader and make it more difficult to fully grasp the research content. Additionally, some sections would benefit from further clarification, as certain parts are challenging to follow in terms of logical flow. A thorough review of the manuscript is encouraged to enhance consistency and improve readability. | Thank you for your positive feedback on the  manuscript. Your encouragement is greatly appreciated.  Thank you for your thoughtful review and constructive feedback. We have thoroughly reviewed the manuscript and have clarified the terms of “patients, young breast cancer couples, and parents” and added detailed information to enhance research content and logical flow. |  |
| **Introduction** 1. The introductory section references relevant studies; however, the discussion on prior research concerning communication between cancer patients and their spouses is somewhat brief. Expanding on the research findings and identifying gaps in previous studies is recommended, as this will also help clarify the research objectives | Thank you for your advice.  We have expanded the research findings and identified gaps in previous studies. Refer to section of Introduction. | Page 2-4 |
| 2. The epidemiological description of breast cancer at the beginning of the Introduction is too much information, so I suggest you focus on what you want to assert. | Thank you for your valuable advice.  We have simplified the epidemiological description of breast cancer and expanded the research findings and identified gaps in previous studies. Refer to section of Introduction. | Page 2-4 |
| 3. It is difficult to understand the link between lower breast cancer incidence rates, delayed childbearing age, two or more children policy, and the upbringing of minors with breast cancer. We recommend that you cite the supporting papers and explain them clearly. | We are grateful for the suggestion. According to your advice, we have cite the supporting papers and explain them clearly:  As breast cancer increasingly affects younger individuals and survival rates improve, a growing number of breast cancer patients find themselves parenting underage children (children under the age of 18) ^2^.  Reference:  2. Ghofrani, M., et al., Levels of unmet needs among adolescents and young adults (AYAs) impacted by parental cancer. Support Care Cancer, 2019. 27(1): p. 175-182. | Page 2, see in Introduction section, paragraph 1 |
| 4. The section describing Chinese culture could be made more accessible to readers by including a comparison with other regions or countries and supporting the discussion with relevant citations or other evidence. | Thank you for your valuable advice.We have added comparison with other regions or countries and added the supporting citation: Compared to the emphasis on cultivating children's independence in other culture^1^, in China., due to the long-term influence of Confucian culture that prioritizes family, parents attach great importance to family ties and kinship and have a stronger sense of responsibility in raising children^5^.  Reference:  Lanjekar, P.D., et al., The Effect of Parenting and the Parent-Child Relationship on a Child's Cognitive Development: A Literature Review. Cureus, 2022. 14(10): p. e30574. | Page 2, see in Introduction section, paragraph 2 |
| 5. It may be a leap of logic to conclude that the negative effects of depression hinder rehabilitation and quality of life. Based on the aforementioned statements, it is unlikely that it is an impediment to rehabilitation. In addition, please present any articles that support this finding or consider removing the sentence “It can be~”. | Thank you for your valuable feedback.  We have removed the sentence “It can be seen that PC has become an obstacle to the rehabilitation and quality of life of young breast cancer patients.” |  |
| **Methods** 1. Providing a detailed description of the research methods enhances the scientific rigor of qualitative research. It is recommended to elaborate on the methods, as the current simplified description raises questions regarding its clarity and comprehensiveness. | Thank you for your valuable feedback. We appreciate your feedback regarding the Methods section.  We have added detailed information to describe the research process. | Page 4-6 |
| 2. Please explain the rationale for setting the participant's age as a criterion for inclusion in the study. | Thank you for your comments. We could justify this issue as:  According to the World Health Organization’s (WHO) age classification, individuals aged 15–44 are defined as young people (WHO, 2002). In this study, given that the legal marriage age for women in China is 20 years old, a young cancer patient is defined as a cancer patient aged 20–44. |  |
| 3. Please provide a detailed description of the random sampling procedure. | Thank you for your valuable feedback.  This study adopted purposive sampling method to select interviewees. As suggested by the reviewer, we have added detailed description of the purposive sampling procedure. | Page 4-5 |
| 4. It should be clearly stated that both the participants and their spouses are included in the study. Since the study participants are patients and their spouses, the total number of participants should also be adjusted accordingly. | We sincerely thank the reviewer for careful reading.  We have adjusted the number of participants.  Twenty‐six interviews (13 young breast cancer patients and 13 spouses) of the participants were conducted and included in the analysis of this study. | Page 8-9  See in section of Results and Table 1. |
| 5. The relationship between the interviewer and the participant should be described. In qualitative research, reflexivity should be considered, as the researcher's background and perspective are integral to the research process, with the researcher themselves being considered an instrument of the study. | Thank you for your valuable feedback.  We have added information to describe the relationship between the interviewer and the participant. | Page 6, paragraph 2 |
| 6. The description of when, where, and how the interviews were conducted is currently brief. Providing more detailed information will enhance the credibility of the paper. | Thank you for your valuable feedback.  We have added detailed information to describe the interview process. | Page 4-6 |
| 7. Two terms are used: "interview outline" and "interview guide." I recommend unifying these terms. Alternatively, if they are intended to be used differently, please clarify the distinction between them. | Thank you for your valuable feedback.  We have unified the two terms as "interview outline". |  |
| 8. Please provide a detailed description of the procedure for random sampling.  It should be explicitly stated that both the participants and their spouses are included in the study. | Thank you for your valuable feedback.  We have added detailed information to describe the procedure for purposive sampling, and explicitly stated that both the participants and their spouses are included in the study. | Page 4-5 |
| 9. Since the study involves patients and their spouses, the total number of participants should also be revised accordingly. | Thank you for highlighting this issue.  We have adjusted the number of participants:  Twenty‐six interviews (13 young breast cancer patients and 13 spouses) of the participants were conducted and included in the analysis of this study. | Page 8-9  See in section of Results and Table 1. |
| 10. Please provide the name of the reporting guideline for the paper and add supporting references. | Thank you for your advice. We have added the reporting guideline for the paper and add supporting references.  Reporting was performed in accordance with the Consolidated Criteria for Reporting Qualitative Research (COREQ) reporting guidelines. | Page 4, see in “Design and setting” section. |
| **Results**  1. Although the discussion states that the study is a dyadic exploration of the communication experiences of young breast cancer couples raising their minor children, the results do not include a dyadic analysis. In order to use the term "dyadic," the results should reflect the analysis of the couple pairs. | Thank you for your valuable guidance.  We have re-analyzed the data from "dyadic" perspective, and refined the themes. | Page 10-14, see in Results section. |
| 2. Some terms are defined within the results (e.g., negative communication, avoidance of communication, etc.). It is recommended to provide the relevant literature that defines these terms. | Thank you for your valuable feedback.  We have provided the relevant literatures that defines these terms in results. | Page 10, Page 12, See in section of Results. |
| 3. The results not only describe communication experiences but also indicate future communication intentions. Since "I am going to say it" refers to a future intention, it is unclear whether the participant actually expressed this intention. This distinction should be addressed in the discussion. | We sincerely thank the reviewer for careful reading.  We have proofread the description and revised. | See in section of Results. |
| 4. The distinction between subthemes 1 and 2 of Theme 2 is unclear. Please reconsider whether it is necessary to separate these two subthemes. | We sincerely appreciate the valuable comments. As suggested by the reviewer, we have re-analysis the data and have revised sub-theme 1 of Theme 2.  We have revised “Subtheme 1: avoidance of communication to protect parenting emotions” to “Subtheme 1: Avoid communication to prevent emotional fluctuations”. | Page 12 |
| **Discussion**  The discussion should interpret the results of this study in relation to prior studies. This paper presents minimal engagement with existing literature, making it difficult to establish a clear logical and scientific foundation. Numerous studies have examined communication among couples facing cancer. Incorporating the findings of these studies could provide a deeper understanding by examining the relationships and differences between communication about parenting and communication about cancer. A thorough review of the entire document is recommended to ensure textual consistency, as some explanations are insufficient or lack logical clarity. | Thank you for your thoughtful review and constructive feedback. We have thoroughly reviewed the manuscript and made improvements to address the issues raised in the discussion section.  We have added information to interpret the results of this study in relation to prior studies and added explanations to enhance logical clarity. | Page 14-18, See in the section of Discussion. |
