# Supplementary material for: Navigating parenting challenges: A qualitative study of communication experiences among young Chinese couples facing breast cancer while raising underage children
Source: PLoS One. 2026 Apr 2;21(4):e0345543. doi: 10.1371/journal.pone.0345543 (PMC13046115; doi:10.1371/journal.pone.0345543)
Supplement: Attachment — Submitted filename: Response to Reviewers 1.docx [file pone.0345543.s005.docx]

Prof. Godwin Banafo Akrong

Editor,

PLOS ONE

September 4, 2025

Subject: Revision and resubmission of manuscript / PONE-D-24-43554

Dear Professor, Godwin Banafo Akrong,

Thank you for your letter and the opportunity to revise our paper again. The suggestions offered by the reviewers have been immensely helpful, and we also appreciate the insightful comments on revising the manuscript.

We have tabulated the reviewer’s comments immediately after this letter and responded to them individually, indicating exactly how we addressed each concern or problem and describing the changes we have made. The revisions have been approved by all authors. The changes are marked in **red** in the paper as you requested, and the revised manuscript was uploaded and submitted to the author centre.

We hope the revised manuscript is deemed suitable for the Plos One, and we are happy to consider further revisions if necessary. We look forward to a favourable reply, and we thank you for your continued interest in our research. If we do not meet the modification criteria, please give us another opportunity to communicate and make modifications.

Sincerely,

| **Academic Editor Comments** | **Authors Respond*** | **Section/Page (P) Number** |
| --- | --- | --- |
| In the online submission form you indicate that your data is not available for proprietary reasons and have provided a contact point for accessing this data. Please note that your current contact point is a co-author on this manuscript. According to our Data Policy, the contact point must not be an author on the manuscript and must be an institutional contact, ideally not an individual. Please revise your data statement to a non-author institutional point of contact, such as a data access or ethics committee, and send this to us via return email. Please also include contact information for the third party organization, and please include the full citation of where the data can be found. | Thank you again for highlighting this issue. We have revised the data statement:  Data are available from Figshare: 10.6084/m9.figshare.30050026. | Page 21 |
| **Reviewer #2 Comments** | **Authors Respond*** | **Section/Page (P) Number** |
| **Introduction** Q1. In the Introduction, previous studies on communication between cancer patients and their spouses are reviewed, and the research questions are presented. However, the purpose of the study is not clearly stated. Please clearly articulate the purpose of the study in the main text.  Additionally, what distinguishes the general parenting concerns of cancer patients from those specific to breast cancer patients? The rationale for focusing specifically on breast cancer patients in this study should be clearly explained. | Thank you again for your positive feedback on the manuscript.  We have articulate the purpose of the study in the main text.  Thank you for your advice.  We have explained the rationale for focusing specifically on breast cancer patients in this study. | Page 4  Page 2-3 |
| Q2. “Compared to the emphasis on cultivating children's independence in other culture[7], “in China, due to the long-term influence of Confucian culture that prioritizes family, parents attach great importance to family ties and kinship and have a stronger sense of responsibility in raising children.  This seems to involve a logical leap. While I understand that Chinese families place great importance on child-rearing, other cultures also highly value family and children. In general, when mothers are unable to care for their children due to cancer, it is reasonable to expect that they would experience significant concern and anxiety. A more nuanced and culturally sensitive explanation would strengthen this argument. | Thank you for your valuable guidance.  We have added information to strengthen this argument. | Page 3 |
| **Methods** Q3. (3) parenting at least a child aged ＜ 18 years  The current phrasing is unclear and may be difficult for readers to interpret. If the intended meaning is that the individual is parenting at least one child under 18 years of age, a clearer and more natural expression should be used | Thank you for your valuable feedback.  We have clarified the statement:  (3) currently raising at least one child under the age of 18 | Page 5 |
| Q4. (1) those who are aware of their spouse's condition;  It is unclear who the term “spouse” refers to in this context. If it refers to the breast cancer patient, the wording should be revised to avoid confusion. | We sincerely thank the reviewer for careful reading.  We have clarified the statement:  (1) those who are aware of patient's diagnosis. | Page 5 |
| **Discussion**  Q5. In this study, we have interviewed 13 patients.  You are conducting interviews with 13 couples who are breast cancer patients, right? Please revise the text. | We sincerely thank the reviewer for careful reading.  Yes, we conducted interviews with 13 couples. We have revised in the text. | Page 15 |
| Q6. The influence of Chinese culture might be somewhat overstated in this analysis. Many of the findings in this study appear to reflect experiences and emotional responses that are common across cultures. In particular, patients’ and their spouses’ perceptions and emotional reactions to cancer, as well as their love and concern for their children, are likely to share significant commonalities regardless of cultural background.  Moreover, previous studies have shown that some cancer patients often suppress their own emotions and avoid sharing them with family members in an effort to protect their loved ones. It is recommended that the discussion engage more thoroughly with the existing literature on communication among cancer patients, their spouses, and families. In light of these considerations, it would be appropriate to revise the discussion section.  Additionally, for patients and spouses who are coping with economic insecurity, caregiving responsibilities, childcare, and household burdens, it is worth questioning whether communication support alone is sufficient to address these challenges. It may be worth considering whether adequate formal and informal support systems—both public and private—exist to assist families facing such difficulties. It may be helpful to note the need to consider broader structural and social support issues. | We sincerely appreciate the valuable comments. We have thoroughly reviewed the manuscript and made improvements to address the issues raised in the discussion section.  Thank you for your valuable guidance.  We have added information to illustrate that in addition to improving the communication skills of patients and their spouses, it is also worth considering providing formal or informal support for families with breast cancer. | Page 16-18  Page 19-20 |
