# Supplementary material for: Navigating parenting challenges: A qualitative study of communication experiences among young Chinese couples facing breast cancer while raising underage children
Source: PLoS One. 2026 Apr 2;21(4):e0345543. doi: 10.1371/journal.pone.0345543 (PMC13046115; doi:10.1371/journal.pone.0345543)
Supplement: Attachment — Submitted filename: Response to Reviewers 3.0.docx [file pone.0345543.s006.docx]

Prof. Godwin Banafo Akrong

Editor,

PLOS ONE

December 21, 2025

Subject: Revision and resubmission of manuscript / PONE-D-24-43554R2

Dear Professor, Godwin Banafo Akrong,

Thank you for your letter and the opportunity to revise our paper again. The suggestions offered by Reviewer #4 have been immensely helpful, and we also appreciate the insightful comments on revising the manuscript.

We have tabulated the reviewer’s comments immediately after this letter and responded to them individually, indicating exactly how we addressed each concern or problem and describing the changes we have made. The revisions have been approved by all authors. The changes are marked in **red** in the paper as you requested, and the revised manuscript was uploaded and submitted to the author centre.

We hope the revised manuscript is deemed suitable for the Plos One, and we are happy to consider further revisions if necessary. We look forward to a favourable reply, and we thank you for your continued interest in our research. If we do not meet the modification criteria, please give us another opportunity to communicate and make modifications.

Sincerely,

| **Academic Editor Comments** | **Authors Respond*** | **Section/Page (P) Number** |
| --- | --- | --- |
| Please review your reference list to ensure that it is complete and correct. If you have cited papers that have been retracted, please include the rationale for doing so in the manuscript text, or remove these references and replace them with relevant current references. Any changes to the reference list should be mentioned in the rebuttal letter that accompanies your revised manuscript. If you need to cite a retracted article, indicate the article’s retracted status in the References list and also include a citation and full reference for the retraction notice. | Thank you for highlighting this issue.  We have not cited papers that have been retracted.  We have reviewed the reference list and ensure that it is complete and correct and have made no changes to the reference list. |  |
| **Reviewer #4 Comments** | **Authors Respond*** | **Section/Page (P) Number** |
| While the manuscript is strong, the following points could enhance its impact, clarity, and scholarly contribution  1. Speaking about Chinese culture in the discussion could you strengthen the discussion with deeper culture nuanced, this would bring a nuanced analysis from being culturally specific to being a culturally informed contribution to the global literature on cancer couple communication | Thank you for your valuable guidance.  We have add information to bring a nuanced analysis from being culturally specific to being a culturally informed contribution to the global literature on cancer couple communication. | Page 17-18 |
| 2. Result section: Suggestion for Sub-theme 2.1 ("instrumental support" and emotional avoidance). This is a powerful finding, is it possible to get some quotes hat shows the husband's practical action and the wife's perception of emotional distance in the same interaction. | Thank you for your valuable guidance.  We have added more quotes to show the husband's practical action and the wife's perception of emotional distance in the same interaction. | Page 13 |
