# Supplementary material for: Navigating parenting challenges: A qualitative study of communication experiences among young Chinese couples facing breast cancer while raising underage children
Source: PLoS One. 2026 Apr 2;21(4):e0345543. doi: 10.1371/journal.pone.0345543 (PMC13046115; doi:10.1371/journal.pone.0345543)
Supplement: Attachment — Submitted filename: Response_to_Reviewers_auresp_4.docx [file pone.0345543.s007.docx]

Prof. Godwin Banafo Akrong

Editor,

PLOS ONE

February 14, 2026

Subject: Revision and resubmission of manuscript / PONE-D-24-43554R3

Dear Professor, Godwin Banafo Akrong,

Thank you for your letter and the opportunity to revise our paper again. The suggestions offered by Reviewer #4 have been immensely helpful, and we also appreciate the insightful comments on revising the manuscript.

We have tabulated the reviewer’s comments immediately after this letter and responded to them individually, indicating exactly how we addressed each concern or problem and describing the changes we have made. The revisions have been approved by all authors. The changes are marked in **red** in the paper, and the revised manuscript was uploaded and submitted to the author centre.

We hope the revised manuscript is deemed suitable for the Plos One, and we are happy to consider further revisions if necessary. We look forward to a favourable reply, and we thank you for your continued interest in our research. If we do not meet the modification criteria, please give us another opportunity to communicate and make modifications.

Sincerely,

| **Academic Editor Comments** | **Authors Respond*** | **Section/Page (P) Number** |
| --- | --- | --- |
| Please review your reference list to ensure that it is complete and correct. If you have cited papers that have been retracted, please include the rationale for doing so in the manuscript text, or remove these references and replace them with relevant current references. Any changes to the reference list should be mentioned in the rebuttal letter that accompanies your revised manuscript. If you need to cite a retracted article, indicate the article’s retracted status in the References list and also include a citation and full reference for the retraction notice. | Thank you for highlighting this issue.  We have not cited papers that have been retracted.  We have reviewed the reference list and ensure that it is complete and correct and have made no changes to the reference list. |  |
| **Reviewer #4 Comments** | **Authors Respond*** | **Section/Page (P) Number** |
| 1. Can the authors clarify why they chose to conduct the study from the Yibin Second People’s Hospital? | Thank you again for your positive feedback  The selection of Yibin Second People's Hospital to conduct this study was based on the following considerations:  Yibin Second People’s Hospital is situated at the junction of Sichuan, Yunnan, and Guizhou provinces in China. This unique geographical location naturally positions it as a regional medical center, serving a highly diverse patient population from different provinces. Such diversity is reflected not only in geographical origins but also profoundly in patients' ethnic composition, dialect and cultural backgrounds, socioeconomic status, health beliefs, and healthcare-seeking behaviors. For this study, this sample characteristic significantly enhances the richness and representativeness of the research. It enables us to explore, within an authentic and socio-culturally diverse context, the common challenges and differentiated coping strategies faced by young couples with breast cancer while raising children, thereby yielding findings with greater generalizability and cultural sensitivity.  Additionally, in terms of research feasibility, the researcher's employment at Yibin Second People's Hospital provided irreplaceable advantages in coordinating resources, complying with ethical protocols, and efficiently recruiting eligible participants. Additionally, the researcher's familiarity with the hospital environment and the local patient population greatly facilitated the establishment of trust, thereby laying a solid foundation for collecting in-depth and authentic qualitative data. |  |
| 2. The authors provided enough demographic information on the participants, however, in reporting the quotes made by the participants the authors did not support them with these demographics to help readers appreciate the findings. The authors can report it as follows (S11, 40 years, staff) OR (P11, 35 years, 2 children, Period III) | Thank you once again for your careful review and constructive feedback.  In the revised manuscript, we have uniformly updated the citation format for all participant quotes, adopting the format suggested by the reviewer or supplementing with more contextually relevant details as appropriate. | Page 12-16 |
| 3. The implications of the study should clearly be presented by the authors. | Thank you once again for your valuable guidance.  We have expanded the information to provide further details regarding the implications. | Page 22 |
| 4. Although the authors mention purposive sampling with maximum variation, they did not provide detailed information on how diversity in education level, cancer stage, and other demographic factors were ensured during participant recruitment. | Thank you once again for your valuable guidance.  We have supplemented the explanation of the specific operational approach for ensuring sample diversity through stratified sampling in the **Method** section: Participants were recruited based on cancer stage (I–III), education level (high school or below / college / undergraduate /postgraduate), parental status (number and age of children), and family residence (city/town). During the recruitment process, the research team reviewed the distribution of included samples on a weekly basis and dynamically adjusted subsequent recruitment priorities until theoretical saturation was achieved across all dimensions, with no new characteristic patterns emerging. Through these steps, the final sample of 13 couples in this study demonstrated significant variation across the key dimensions outlined, aligning with the design objective of maximum variation sampling. | Page 5 |
| 5. The authors state that data saturation was achieved after interviewing 13 couples, however, can the authors provide a clear explanation of how saturation was determined. Including specific criteria. | Thank the reviewer for the important comments regarding the data saturation of our study.  The sample size was determined by data saturation, which was achieved when no new themes emerged from the interviews and the assess method from Guest [25]. In this study, no new themes emerged after the 11th interview. According to the method proposed by Guest, a total of 21 new themes were generated from interviews 1 to 9, while only one new theme was identified from interviews 10 to 11. This represented an incremental yield of 4.7% (1/21), which fell below the predetermined saturation threshold of 5%, indicating that saturation was achieved. Two additional interviews were then conducted, and no new themes were observed. By the 13th interview, the principle of maximum participant variation was satisfied. Therefore, a final sample of 13 participants was included in this study.  Reference:  *Guest, G., E. Namey, and M. Chen, A simple method to assess and report thematic saturation in qualitative research. PLoS One, 2020. 15(5): p. e0232076.* | Page 5-6 |
| 6. Since the authors mention that the interviewer played dual roles as an “insider” and “outsider,” can the authors elaborate on how potential bias from the interviewer’s prior relationship with patients was mitigated? | We sincerely appreciate the reviewer’s important comments on the data collection of our study. We have added information about how potential bias from the interviewer’s prior relationship with patients was mitigated:  To mitigate potential bias arising from the interviewer’s dual role as both an “insider” and “outsider,” several strategies were implemented. First, the semi-structured interview guide was collaboratively developed and reviewed by external experts to ensure neutrality and openness in questioning. Second, during interviews, the interviewer adopted an “empathic neutrality” approach, balancing rapport-building with professional detachment, and used probing techniques to explore participants’ unique experiences rather than relying on prior familiarity. Third, investigator triangulation was employed, with at least two researchers conducting independent data analysis followed by team discussions to challenge interpretations and minimize individual bias. Finally, member checking with a subset of participants verified the accuracy and resonance of the findings.  Thank you again for your constructive feedback, which has helped strengthen the methodological rigor of our study. | Page 9 |
| 7. Can the authors provide more information on how codes were developed and refined, as well as the process of resolving discrepancies between researchers. This is to help address the issues of transparency and replicability. | Thank you for your valuable comment regarding the need for more detailed information on code development, refinement, and the process of resolving discrepancies between researchers. We appreciate the opportunity to enhance the transparency and replicability of our analytical process.  We have expanded the "Data Analysis" section to provide a more comprehensive description of our coding procedures and consensus-building process.  Thank you again for your constructive feedback, which has helped strengthen the methodological transparency of our study. | Page 8-9 |
